# Alcohol and aging: Next‐generation epigenetic clocks predict biological age acceleration in individuals with alcohol use disorder

**DOI:** 10.1111/acer.70020

**Published:** 2025-03-28

**Authors:** Tyler A. Perlstein, Jeesun Jung, Alexandra C. Wagner, Joshua Reitz, Josephin Wagner, Daniel B. Rosoff, Falk W. Lohoff

**Affiliations:** ^1^ Section on Clinical Genomics and Experimental Therapeutics National Institute on Alcohol Abuse and Alcoholism, National Institutes of Health Bethesda Maryland USA; ^2^ NIH Oxford‐Cambridge Scholars Program, Radcliffe Department of Medicine University of Oxford Oxford UK

**Keywords:** alcohol consumption, alcohol use disorder, DNA methylation clock, epigenetic aging

## Abstract

**Background:**

Chronic heavy alcohol use is a major risk factor for premature aging and age‐related diseases. DNA methylation (DNAm)‐based epigenetic clocks are novel tools for predicting biological age. However, the newest configurations, causality‐enriched epigenetic clocks, have not been assessed in the context of alcohol consumption and alcohol use disorder (AUD).

**Methods:**

Epigenetic aging was evaluated in a sample of 615 individuals (372 AUD patients and 243 healthy controls) by applying the GrimAge Version 1 (V1) and Version 2 (V2) clocks alongside three causality‐enriched clocks (CausAge, DamAge, and AdaptAge). A linear model controlling for AUD diagnosis, sex, race, BMI, smoking status, and five blood cell types was leveraged to test associations between alcohol‐related metrics and age‐adjusted epigenetic clocks.

**Results:**

GrimAge V1 and V2 maintained significant associations with AUD and drinking behavior measures within the total sample and both the young (<40 years old) and old (≥40 years old) subgroups. Generally, GrimAge V2 slightly outperformed GrimAge V1, while none of the causality‐enriched epigenetic clocks demonstrated significant associations with AUD. However, in the young subgroup, DamAge had a significant association with the total number of drinks. Across the total sample and age subgroups, with liver function enzymes, GrimAge V2 consistently sustained stronger associations compared with GrimAge V1. Among fourth‐generation clocks, DamAge exhibited significant associations with gamma‐glutamyl transferase (GGT) and aspartate aminotransferase in the total sample and young subgroup; CausAge displayed a significant association with GGT in the total sample. Examining clinical biomarkers, GrimAge V2 showed improved associations with C‐reactive protein compared to GrimAge V1 in the total sample and age subgroups.

**Conclusions:**

Overall, we observed moderately improved performance of GrimAge V2 compared with GrimAge V1 with the majority of the parameters tested. The causality‐enriched epigenetic clocks lacked significant associations but demonstrate the complexities of aging and inspire further research of AUD and drinking dynamics.

## Graphical abstract

Our study investigated the performance of five epigenetic clocks by evaluating associations with alcohol use disorder (AUD) and various alcohol‐related phenotypes. Both DNAm GrimAge versions were associated with AUD and alcohol behaviors/clinical measures in the total sample and age subgroups. Generally, GrimAge V2 moderately outperformed V1, whereas the causality‐enriched clocks—CausAge, DamAge, and AdaptAge—lacked significant associations. These results highlight the predictive potentials of hemoglobin A1C and C‐reactive protein, both incorporated in GrimAge V2, and invite further exploration of these algorithms.

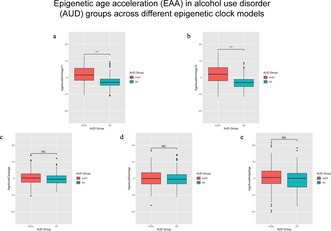

## INTRODUCTION

Alcohol misuse is a leading cause of early mortality, resulting in 7.2% of all premature deaths annually (World Health Organization, 2018). Alcohol use disorder (AUD) is characterized by excessive and persistent consumption of alcohol that results in serious clinical impairment and distress. Previous studies indicate that a history of AUD is fundamentally linked to a heightened risk for chronic health conditions and premature death, especially in the elderly population (Leong et al., 2022; Udo et al., 2015). Specifically, compared with healthy control (HC) individuals, AUD patients are at higher risk for developing aging‐related diseases such as cancer, diabetes, dementia, depression, cardiovascular conditions, and digestive disorders (Saieva et al., 2012; Schwarzinger et al., 2017; Sullivan & Pfefferbaum, 2019; Vancampfort et al., 2016). These findings are reflective of accelerated aging among AUD patients and emphasize the urgency in further investigating the effects of alcohol on aging and how it alters different mechanisms within the body (Sullivan & Pfefferbaum, 2019).

To better understand the impact of alcohol on the aging process, geroscience research has applied methylation‐based proxy models to predict biological age. These models are referred to as *epigenetic clocks* (Hannum et al., 2013; Horvath, 2013; Levine et al., 2018; Lu et al., 2019, 2022). Epigenetic clocks offer insight into biological aging through their derivation from methylation signatures (López‐Otín et al., 2013) and are built upon a unique framework of age‐associated cytosine phosphate guanine (CpG) sites, where the DNA methylation (DNAm) ages are computed as the weighted average of the methylation levels at these specific sites (Hannum et al., 2013; Horvath, 2013; Levine et al., 2018; Lu et al., 2019, 2022). Not only are these clocks significantly associated with chronological age, but they also reveal the nuances and sensitive properties of methylome modification (Oblak et al., 2021). Certain CpG sites remain relatively stable while others fluctuate more drastically over time, and these patterns express cumulative effects of the aging process (Jung et al., 2023). Epigenetic modifications can result from different environmental exposures, such as sources of physiological and psychological stress (Ashapkin et al., 2017). Therefore, epigenetic clocks offer a novel approach to studying the effects of AUD on underlying mechanisms of aging (Lohoff et al., 2021; Longley et al., 2021).

The construction of each epigenetic clock is based upon the aggregation of methylome measures. The first‐generation epigenetic clocks (i.e., Horvath's DNAm age [HorvathAge] and Hannum's DNAm age [HannumAge]) predict chronological age from methylation data collected from earlier versions of the Illumina methylation arrays (27K and 450K chips) (Hannum et al., 2013; Horvath, 2013). The second‐generation epigenetic clocks, namely DNAm PhenoAge and GrimAge Version 1 (GrimAge V1) and GrimAge Version 2 (GrimAge V2), predict mortality risk by incorporating additional age‐related biomarkers (Levine et al., 2018; Lu et al., 2019, 2022). The singular third‐generation epigenetic clock, DunedinPace, estimates a pace of epigenetic aging that indicates the rate of change in epigenetic age (Belsky et al., 2022). The fourth‐generation epigenetic clocks are the most recently released and have introduced an element of causality in approximating biological age (Ying et al., 2024).

Scientific literature has shown conflicting findings regarding the association of alcohol consumption with biological age across different epigenetic clocks and demographic groups. One of the earliest studies performed did not uncover a significant effect of alcohol consumption on the HannumAge in African American females (*N* = 100) (Simons et al., 2016). Fiorito et al. (2019) performed a multicohort analysis (*N* = 16,245) demonstrating that PhenoAge upheld a more significant association with alcohol use in comparison to HorvathAge and HannumAge (*p*‐value (*p*) < 0.001). In addition, PhenoAge was 2.51 years advanced in the heavy alcohol use group (*p* < 0.05) in the UK Airwave cohort (*N* = 2239) (Robinson et al., 2020). Examining the Multi‐Ethnic Study of Atherosclerosis (MESA) (*N* = 1211) and Health and Retirement Study (HRS) (*N* = 4018) datasets, neither the first nor second‐generation epigenetic clocks illustrated significant associations with alcohol consumption (Schmitz et al., 2022). Our studies with a mixed demographic cohort previously detected epigenetic age acceleration of 2.22 years as captured by PhenoAge in AUD patients compared with HCs (Luo et al., 2020). They have also shown heavy alcohol use to be significantly related to second‐generation epigenetic clocks, particularly GrimAge V1 as well as DNAm telomere length (DNAmTL) (Jung et al., 2022, 2023).

Although numerous studies have included GrimAge V1 in their analyses, few have incorporated the most up‐to‐date version of the clock, GrimAge V2. Version 1 aggregates seven DNAm‐based proxies of plasma proteins, a DNAm‐based proxy of smoking pack‐years (one pack‐year is equal to one pack of cigarettes smoked per day for a year), age, and sex (Lu et al., 2019). Alongside these biomarkers, GrimAge V2 additionally integrates DNAm‐based proxies of log‐transformed high sensitivity C‐reactive protein (logCRP) and hemoglobin A1C (logA1C) (Lu et al., 2022). Lu et al. (2022) have demonstrated that GrimAge V2 outclasses GrimAge V1 in mortality prediction across multiple racial groups (GrimAge V2 *p* = 3.6E‐167, GrimAge V1 *p* = 2.6E‐144) and its associations with different age‐related conditions. Incorporating an epigenome‐wide Mendelian randomization (EWMR) approach, the modern fourth‐generation epigenetic clock models designed by Ying et al. (2024) adopt strictly causal cytosine phosphate guanine (CpG) localities. By separating causal CpGs into those that are harmful and those that are protective during aging by observing age‐associated outcomes, three new clocks were developed: DamAge (encompassing only deleterious CpG sites), AdaptAge (boasting only beneficial CpG sites), and CausAge (incorporating all causative CpG sites) (Ying et al., 2024). These three clocks were reported to demonstrate significant associations with various age‐related diseases.

The newly developed epigenetic clocks capture various clinical characteristics of biological aging, but little has been studied about how alcohol misuse comes into play in these new models. In order to evaluate the impact of alcohol on aging, we investigated five recently released epigenetic clocks (GrimAge V1, GrimAge V2, CausAge, DamAge, and AdaptAge) and compared their associations using a broad range of alcohol consumption‐related measures and clinical biomarkers.

## MATERIALS AND METHODS

The sample consisted of 615 participants, 372 with AUD and 243 HCs, recruited at the National Institute on Alcohol Abuse and Alcoholism (NIAAA) at the National Institutes of Health (NIH) in the United States of America. Participants were recruited through the NIAAA Natural History Protocol, a study aiming to screen different characteristics of treatment and non‐treatment seeking individuals with the goals of enhancing the general understanding of AUD and configuring placement in other research studies at the NIH. Inclusion criteria included being 18 years of age or older and a desire to finish the study and all related clinical tests by providing a blood sample. Exclusion criteria included being less than 18 years of age, being pregnant, being incarcerated, or being diagnosed with severe medical conditions or mental disorders that would interfere with study participation. Participants completed a phone screening to assess their eligibility, and those who were eligible provided blood samples, completed questionnaires, and underwent evaluation of their health and alcohol consumption behaviors. In addition, those seeking treatment were assessed before and after placement in either outpatient, inpatient, or both treatment programs. More information about the study can be found at this link: https://clinicalstudies.info.nih.gov/ProtocolDetails.aspx?id=2014‐AA‐0181#summary. All study participants completed the Structured Clinical Interview for DSM‐IV (Diagnostic and Statistical Manual of Mental Disorders, fourth edition) (SCID) to determine an alcohol dependence (AD) diagnosis (American Psychiatric Association, n.d.). Given the overlap between the DSM‐IV AD criteria and the DSM‐5 AUD criteria, all participants with AD also met criteria for AUD diagnosis (Goldstein et al., 2015). Participants provided a blood sample that was used for genome‐wide DNA methylation analysis and clinical biomarker collection, including liver function enzymes (LFEs) such as gamma‐glutamyl transferase (GGT), alanine aminotransferase (ALT), and aspartate aminotransferase (AST) as well as C‐reactive protein (CRP; *N* = 423) and hemoglobin A1C (*N* = 339). Participants also completed self‐report questionnaires, including the Fagerström Test for Nicotine Dependence (FTND), a metric reflective of smoking habits (Heatherton et al., 1991), and the Timeline Followback (TLFB) questionnaire (Sobell & Sobell, 1992) to measure the number of alcoholic drinks consumed per day for the previous 90 days, in units of standard drinks. The total number of drinks, the number of drinking days, the average number of drinks per drinking day, and the number of heavy drinking days variables were measured based on the 90‐day TLFB analysis. A heavy drinking day is defined based on the NIAAA definition as ≥4 drinks per day for women and ≥5 drinks per day for men. All study participants provided written informed consent in accordance with the Declaration of Helsinki and were compensated for their time. The study was approved by the Institutional Review Board (IRB) of the NIH.

### DNA methylation measurements and estimation of DNAm GrimAges

DNA methylation levels from whole blood samples were assessed using an Infinium MethylationEPIC BeadChip microarray (manufactured by Illumina Inc., San Diego, CA, USA). The wateRmelon package in R was used to process the raw data. Cross‐reactive probes and probes that failed quality assessment were removed, and then a scale‐based correction was applied to Illumina type I probes relative to type II probes. A quantile normalization approach was executed for standardizing methylated and unmethylated allele intensities, and then *β*‐values were calculated using the ratio of intensities. Estimates of blood cell proportions of six cell types (monocytes, granulocytes, natural killer cells, B cells, CD4+ T cells, and CD8+ T cells) were generated using the Houseman estimation method (Houseman et al., 2016). GrimAge V1 and GrimAge V2 were calculated using Horvath's epigenetic age calculator software (https://dnamage.clockfoundation.org/).

### Estimation of CausAge, DamAge, and AdaptAge

The causal epigenetic clock models (CausAge, AdaptAge, and DamAge), developed with an epigenome‐wide Mendelian randomization (EWMR) approach and reliance on age‐associated differential methylation, offer distinguishments of helpful changes from harmful ones (Ying et al., 2024). CausAge was estimated through 586 CpGs selected by a causality‐enriched elastic net model. DamAge contains only damaging CpG sites (*n* = 1090) and estimates higher mortality risk in individuals expected to obtain more harmful changes during aging. Meanwhile, AdaptAge consists of only adaptive CpGs (*n* = 1000) and predicts lower mortality risk in individuals expected to accumulate more protective changes during aging (Ying et al., 2024). In other words, a higher AdaptAge value represents the aggregation of more beneficial changes and presumably enhanced longevity, while a greater DamAge value represents the accrual of more deleterious changes and presumably degraded longevity (Ying et al., 2024). In our sample, we utilized 396 CausAge CpGs, 1089 DamAge CpGs, and 998 AdaptAge CpGs. There were some discrepancies in the CpGs available to implement due to the clocks being trained on the Illumina 450K chip platform. Nevertheless, by integrating the weight of each available CpG, we computed causality‐enriched epigenetic ages as represented by CausAge, DamAge, and AdaptAge.

### Statistical analysis

To investigate differences in age‐adjusted GrimAge V1, GrimAgeV2, CausAge, DamAge, and AdaptAge between AUD and HC cohorts, a linear model was utilized with adjustment for the covariates sex, race, body mass index (BMI), smoking status, and estimated proportions of five blood cell types (monocytes, natural killer cells, B cells, CD4+ T cells, and CD8+ T cells). Granulocytes were not included in the model following the execution of variance inflation factor analysis (Jung et al., 2023). The age‐adjusted epigenetic clocks (defined by epigenetic age acceleration, EAA) were calculated by taking the residual resulting from a linear regression of the DNAm age on chronological age. Furthermore, we additionally controlled for AUD diagnosis to test associations with alcohol consumption behaviors, LFE biomarkers (i.e., GGT, AST, ALT), and alternate clinical biomarkers (i.e., A1C, CRP). We first conducted our analyses with the total sample (*N* = 615) and then divided the total sample into a young cohort (<40 years old) and an old cohort (≥40 years old) to investigate differences across generations. We tested to see whether there was an interaction effect between AUD diagnosis (AUD vs. HC) and age group (young vs. old) in the linear model; subsequent analyses within each age subgroup were carried out once a significant interaction effect between AUD diagnosis and age group was observed. All alcohol‐related clinical variables were standardized (mean = 0, SD = 1) to generate interpretable results across a wide spectrum of scales. There was no evidence to suggest a non‐Gaussian distribution of the residuals from the models (Shapiro–Wilk test, *p* > 0.05).

There may be potential systematic differences between the AUD and HC groups in terms of several confounding covariates (e.g., smoking status). To control for bias in the comparison of these two groups, propensity scores for retrospective matching analysis were calculated (Chen et al., 2022). With the total sample (372 AUD and 243 HC participants), a propensity score was generated for each participant using a logistic regression model with the same covariates (age, sex, race, BMI, smoking status, five blood cell compositions). Based on these propensity scores, the optimal approach with a greedy matching algorithm matched 243 AUD patients and 243 HC individuals (1:1 matching), which left 129 AUD patients unmatched. The balance in the matched group was assessed before and after matching. Using the matched sample, the differences in epigenetic age acceleration (EAA) between the two groups as well as the associations with the alcohol consumption‐based measures were tested after adjusting for propensity scores.

Finally, a formal statistical test was carried out to investigate whether there is a significant difference in the effect size of age‐adjusted GrimAge V1 and V2, in which the models were tested with the difference between age‐adjusted DNAm age as the dependent variable and the alcohol use measure of interest as the independent variable with adjustment for the covariates mentioned above (the theoretical formula is available in Appendix S1). All statistical significance was defined by a Bonferroni‐corrected significance level (0.01 = 0.05/5, testing five epigenetic clocks). Statistical analyses were performed in R version 4.2.

## RESULTS

Study participant demographic characteristics are described in Table 1. Participants with AUD and HC individuals differed significantly in chronological age (*p* < 0.0001), where AUD cases were typically older, with a mean age of 44.52 years (Table 1). AUD cases had a higher proportion of males (60.22%, *p* = 0.005) and smokers (62.63%, *p* < 0.0001) compared with HC individuals, but neither group strongly differed in race/ancestry distribution (Table 1). Individuals with AUD generally had an increased BMI, elevated LFEs, and more severe alcohol consumption behaviors (volume and frequency) than those in the HC group (*p*s < 0.01) (Table 1).

**TABLE 1:** Sociodemographic characteristics of study participants.

	HC (*N* = 243)		AUD (*N* = 372)		*p*‐value
	*N*	%	*N*	%	
Sex					
Male	118	48.56	224	60.22	0.005
Race					
European American	133	54.73	191	51.34	0.14
African American	103	42.39	177	47.58	
Other	7	2.88	4	1.08	
Smoking	13	5.35	233	62.63	<0.0001
	**Mean**	**SD**	**Mean**	**SD**	
Age	35.41	12.52	44.52	10.99	<0.0001
BMI	26.31	4.74	27.45	5.81	0.008
Total number of drinks	51.33	113.1	937.52	700.0	<0.0001
Number of drinking days	18.58	21.36	72.95	21.19	<0.0001
Average number of drinks per day	2.16	1.57	12.62	8.01	<0.0001
Number of heavy drinking days	3.51	11.88	64.08	27.41	<0.0001
GGT	26.05	27.66	141.50	235.23	<0.0001
AST	21.39	15.74	53.70	64.14	<0.0001
ALT	23.54	19.18	50.32	54.05	<0.0001
CRP	2.77	1.97	5.06	11.72	0.001
A1C	5.30	0.50	5.38	0.53	0.19

*Note*: Heavy drinking days are defined as greater than or equal to 4 drinks a day for females and greater than or equal to 5 drinks a day for males. *p*‐values were obtained through two‐sample *t*‐tests.

Abbreviations: A1C, hemoglobin A1C; ALT, alanine aminotransferase; AST, aspartate aminotransferase; AUD, alcohol use disorder; CRP, C‐reactive protein; GGT, gamma‐glutamyl transferase; V1, Version 1; V2, Version 2.

### Associations of AUD and alcohol consumption‐related measures

**FIGURE 1:**
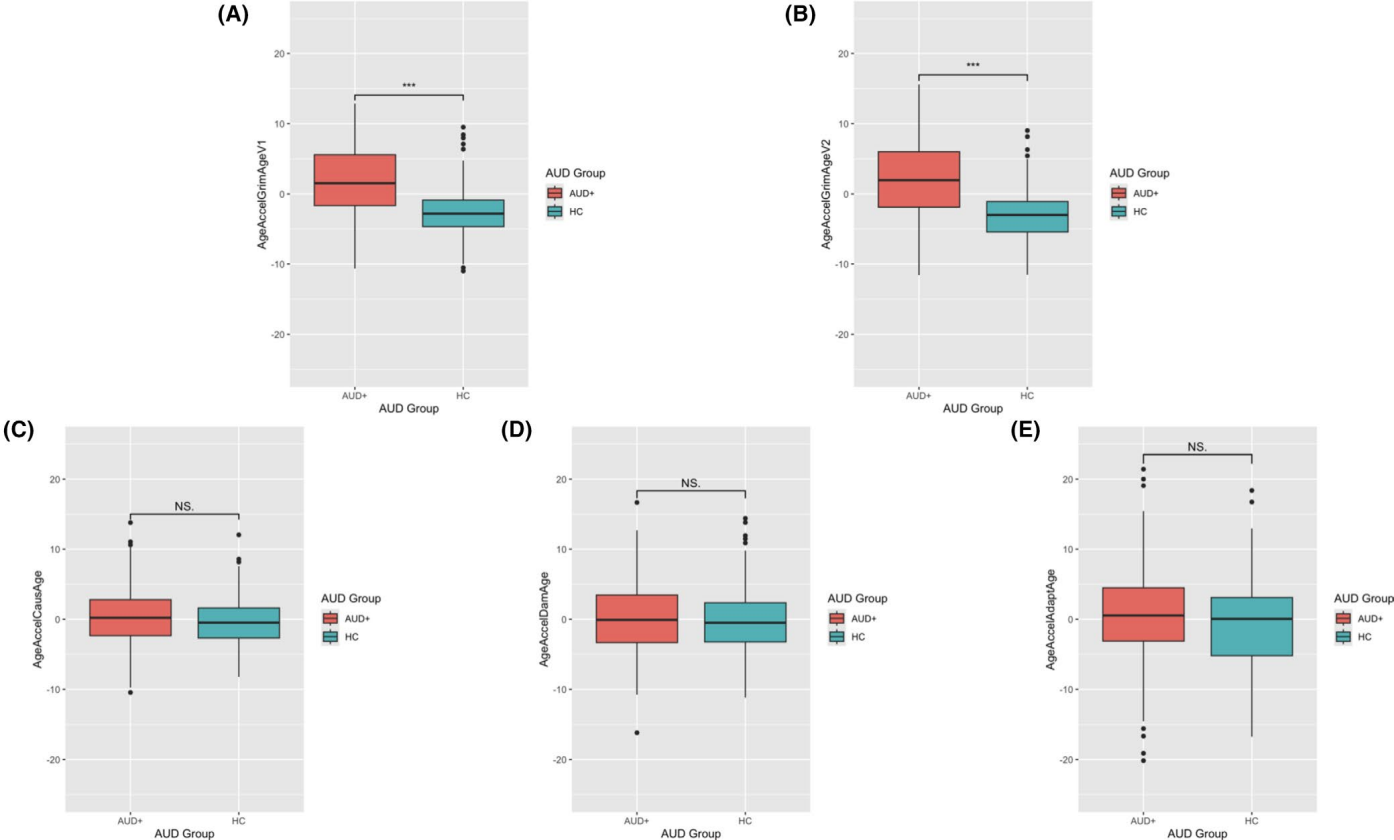
Boxplots illustrating epigenetic age acceleration (EAA) in alcohol use disorder (AUD) groups across different epigenetic clock models without adjustment for covariates. (A) EAA of DNAm GrimAge Version 1 (V1); (B) EAA of DNAm GrimAge Version 2 (V2); (C) EAA of CausAge; (D) EAA of DamAge; (E) EAA of AdaptAge. AUD, alcohol use disorder; EAA, epigenetic age acceleration; HC, healthy control. Significance codes are as follows: “***” when *p*‐value (*p*) < 0.0001, “**” when *p* < 0.001, “*” when *p* < 0.01, “·” when *p* < 0.05, ‘NS’ (not significant) otherwise. *p*‐values were obtained through two sample *t*‐tests.

We performed analyses modeling the relationship between alcohol consumption‐related metrics and EAA calculated from both versions of GrimAge, CausAge, DamAge, and AdaptAge. Comparing the two GrimAge clocks, we first found AUD status to be significantly associated with GrimAge V1 (*β* = 2.01, *p* = 3.70E‐08) and GrimAge V2 (*β* = 2.43, *p* = 1.50E‐09) (Table 2, Figure 1). In other words, GrimAge V1 was 2.01 years advanced, and GrimAge V2 was 2.43 years advanced in the AUD group as compared to the HC group. However, the causality‐enriched clocks (CausAge, DamAge, and AdaptAge) did not show any statistically significant differences in EAA between AUD patients and HC individuals (Table 2, Figure 1). Further statistical testing suggested a lack of statistically significant difference in the effect sizes of AUD between GrimAge V1 and V2 (Table S1).

**TABLE 2:** Associations of epigenetic clocks with alcohol consumption‐based biomarkers in the total sample (*N* = 615).

Model	Alcohol variable	*β*	SE	*T*	*p*‐value	Significance
GrimAge V1	AUD status	2.01	0.36	5.58	3.70E‐08	***
GrimAge V2	AUD status	2.43	0.40	6.14	1.50E‐09	***
CausAge	AUD status	0.37	0.37	1.00	0.32	
DamAge	AUD status	0.60	0.52	1.14	0.25	
AdaptAge	AUD status	0.91	0.64	1.43	0.15	
GrimAge V1	Total number of drinks	0.82	0.17	4.75	2.56E‐06	***
GrimAge V2	Total number of drinks	0.95	0.19	5.02	7.00E‐07	***
CausAge	Total number of drinks	0.30	0.18	1.65	0.10	
DamAge	Total number of drinks	0.47	0.25	1.84	0.07	
AdaptAge	Total number of drinks	−0.13	0.31	−0.42	0.67	
GrimAge V1	Number of drinking days	0.23	0.22	1.04	0.30	
GrimAge V2	Number of drinking days	0.26	0.24	1.09	0.28	
CausAge	Number of drinking days	0.23	0.23	1.01	0.32	
DamAge	Number of drinking days	−0.004	0.32	−0.01	0.99	
AdaptAge	Number of drinking days	0.62	0.39	1.62	0.11	
GrimAge V1	Average number of drinks per day	0.78	0.18	4.41	1.25E‐05	***
GrimAge V2	Average number of drinks per day	0.87	0.20	4.47	9.47E‐06	***
CausAge	Average number of drinks per day	0.31	0.19	1.66	0.10	
DamAge	Average number of drinks per day	0.53	0.26	2.05	0.04	·
AdaptAge	Average number of drinks per day	−0.30	0.32	−0.94	0.35	
GrimAge V1	Number of heavy drinking days	1.07	0.22	4.80	1.99E‐06	***
GrimAge V2	Number of heavy drinking days	1.17	0.24	4.77	2.30E‐06	***
CausAge	Number of heavy drinking days	0.26	0.24	1.09	0.27	
DamAge	Number of heavy drinking days	0.51	0.33	1.55	0.12	
AdaptAge	Number of heavy drinking days	0.09	0.40	0.23	0.82	
GrimAge V1	GGT	0.55	0.14	3.97	8.19E‐05	***
GrimAge V2	GGT	0.76	0.15	5.04	6.05E‐07	***
CausAge	GGT	0.40	0.14	2.81	5.06E‐03	*
DamAge	GGT	0.79	0.20	3.97	7.99E‐05	***
AdaptAge	GGT	0.19	0.25	0.77	0.44	
GrimAge V1	AST	0.47	0.14	3.37	7.91E‐04	**
GrimAge V2	AST	0.67	0.15	4.39	1.32E‐05	***
CausAge	AST	0.26	0.15	1.80	0.07	
DamAge	AST	0.78	0.20	3.84	1.39E‐04	**
AdaptAge	AST	−0.14	0.25	−0.56	0.58	
GrimAge V1	ALT	0.36	0.14	2.51	0.01	·
GrimAge V2	ALT	0.53	0.16	3.36	8.42E‐04	**
CausAge	ALT	0.14	0.15	0.91	0.36	
DamAge	ALT	0.27	0.21	1.29	0.20	
AdaptAge	ALT	0.36	0.25	1.43	0.15	
GrimAge V1	CRP	0.64	0.17	3.89	1.18E‐04	**
GrimAge V2	CRP	0.77	0.18	4.23	2.91E‐05	***
CausAge	CRP	−0.17	0.17	−0.99	0.32	
DamAge	CRP	0.03	0.24	0.12	0.91	
AdaptAge	CRP	0.32	0.29	1.13	0.26	
GrimAge V1	A1C	0.41	0.20	2.09	0.04	·
GrimAge V2	A1C	0.40	0.22	1.82	0.07	
CausAge	A1C	0.19	0.20	0.92	0.36	
DamAge	A1C	−0.34	0.29	−1.14	0.25	
AdaptAge	A1C	0.96	0.38	2.55	0.01	·

*Note*: Heavy drinking days are defined as greater than or equal to 4 drinks a day for females and greater than or equal to 5 drinks a day for males. *p*‐values were obtained through two‐sample *t*‐tests.

Abbreviations: A1C, hemoglobin A1C; ALT, alanine aminotransferase; AST, aspartate aminotransferase; AUD, alcohol use disorder; CRP, C‐reactive protein; GGT, gamma‐glutamyl transferase; V1, Version 1; V2, Version 2.

Significance codes are as follows: “***” when *p*‐value (*p*) < 0.0001, “**” when *p* < 0.001, “*” when *p* < 0.01, “·” when *p* < 0.05.

We also found the following alcohol consumption behavioral variables to be significantly associated with both versions of GrimAge: total number of drinks in the past 90 days, average number of drinks per day, and number of heavy drinking days (*p*s < 0.0001) (Table 2). In particular, the total number of drinks in the past 90 days and average drinks per day were more significantly associated with GrimAge V2 than GrimAge V1 given their effect sizes and *p*‐values (Table 2); however, the association with the number of heavy drinking days was slightly more significant for GrimAge V1 (*β* = 1.07, *p* = 1.99E‐06) than GrimAge V2 (*β* = 1.17, *p* = 2.30E‐06) (Table 2). None of the causality‐enriched epigenetic clocks met the significance threshold for the alcohol behavioral variables (*p*s > 0.01) (Table 2). Liver function enzyme levels, as characterized by GGT, AST, and ALT, were identified to be significantly associated with GrimAge V2 (*p*s < 0.001), while GrimAge V1 had significant associations with GGT and AST (*p*s < 0.001) (Table 2). Overall, the effect sizes with their *p*‐values indicate that all three of these variables (GGT, AST, and ALT) were more significantly associated with GrimAge V2 than GrimAge V1 (Table 2). Meanwhile, DamAge had significant associations with the liver enzyme variables GGT and AST (*p*s < 0.001), while CausAge had a significant association with only GGT (*p* < 0.01) (Table 2).

Looking at the alternative clinical variables, both GrimAge clocks had significant associations with CRP, but GrimAge V2 (*β* = 0.77, *p* = 2.91E‐05) had a slightly stronger association than GrimAge V1 (*β* = 0.64, *p* = 1.18E‐04) (Table 2). For A1C, neither GrimAge V1 nor V2 met the significance threshold. The subsequent analyses of comparison in effect sizes across various alcohol consumption measures between GrimAge V1 and V2 showed no statistically significant differences (Table S1). Nonetheless, GrimAge V2 tends to slightly outperform GrimAge V1 in modeling EAA in the context of AUD. CausAge, DamAge, nor AdaptAge met the significance threshold for the alternative clinical variables (*p*s ≥ 0.01) (Table 2).

### Associations of AUD and alcohol‐related measures within age group

We observed an interaction effect on age‐adjusted GrimAge clocks between AUD diagnosis (AUD vs. HC) and age group (young vs. old) and thus conducted association analyses within subgroups of age. In the young (<40 years old) subgroup, GrimAge V2 supported a slightly more significant association with AUD (*β* = 1.99, *p* = 2.67E‐04) than GrimAge V1 (*β* = 1.59, *p* = 2.06E‐03) (Table 3) even though the statistical significance in an effect size difference between them was not supported (Table S1). Regarding alcohol consumption variables, GrimAge V2 maintained a more significant association with total drinks, but GrimAge V1 retained more significant associations with average drinks per day and number of heavy drinking days (*p*s < 0.01) (Table 3). Amongst the causality‐enriched clocks, DamAge established a significant association with total number of drinks (*β* = 1.11, *p* = 5.41E‐03) (Table 3). With respect to the liver function enzymes, only GrimAge V2 had a significant association with GGT (*p* < 0.01) (Table 3). In addition, DamAge exhibited significant associations with GGT and AST (*p*s < 0.01). GrimAge V2 also had a stronger association with CRP (*β* = 0.77, *p* < 0.01) in comparison with GrimAge V1 (*β* = 0.66, *p* = 0.01) (Table 3).

**TABLE 3:** Associations of epigenetic clocks with alcohol consumption‐based biomarkers in the young cohort (<40 years old).

Model	Alcohol variable	*β*	SE	*T*	*p*‐value	Significance
GrimAge V1	AUD status	1.59	0.51	3.11	2.06E‐03	*
GrimAge V2	AUD status	1.99	0.54	3.69	2.67E‐04	**
CausAge	AUD status	0.72	0.52	1.40	0.16	
DamAge	AUD status	1.14	0.76	1.51	0.13	
AdaptAge	AUD status	0.74	0.90	0.82	0.41	
GrimAge V1	Total number of drinks	1.09	0.26	4.14	4.61E‐05	***
GrimAge V2	Total number of drinks	1.18	0.28	4.25	2.98E‐05	***
CausAge	Total number of drinks	0.60	0.27	2.21	0.03	·
DamAge	Total number of drinks	1.11	0.39	2.81	5.41E‐03	*
AdaptAge	Total number of drinks	0.48	0.48	1.01	0.32	
GrimAge V1	Number of drinking days	0.32	0.32	0.99	0.32	
GrimAge V2	Number of drinking days	0.35	0.34	1.03	0.31	
CausAge	Number of drinking days	−0.07	0.33	−0.20	0.84	
DamAge	Number of drinking days	0.47	0.47	0.99	0.32	
AdaptAge	Number of drinking days	0.51	0.57	0.90	0.37	
GrimAge V1	Average number of drinks per day	0.83	0.27	3.05	2.53E‐03	*
GrimAge V2	Average number of drinks per day	0.85	0.29	2.93	3.76E‐03	*
CausAge	Average number of drinks per day	0.57	0.28	2.06	0.04	·
DamAge	Average number of drinks per day	0.87	0.41	2.14	0.03	·
AdaptAge	Average number of drinks per day	0.54	0.49	1.11	0.27	
GrimAge V1	Number of heavy drinking days	1.33	0.35	3.79	1.87E‐04	**
GrimAge V2	Number of heavy drinking days	1.37	0.37	3.67	2.91E‐04	**
CausAge	Number of heavy drinking days	0.37	0.36	1.01	0.31	
DamAge	Number of heavy drinking days	1.17	0.53	2.23	0.03	·
AdaptAge	Number of heavy drinking days	0.55	0.63	0.87	0.39	
GrimAge V1	GGT	0.36	0.19	1.87	0.06	
GrimAge V2	GGT	0.58	0.20	2.85	4.71E‐03	*
CausAge	GGT	0.29	0.20	1.48	0.14	
DamAge	GGT	0.82	0.29	2.85	4.71E‐03	*
AdaptAge	GGT	0.42	0.34	1.23	0.22	
GrimAge V1	AST	0.27	0.20	1.39	0.17	
GrimAge V2	AST	0.42	0.21	2.05	0.04	·
CausAge	AST	0.22	0.20	1.09	0.28	
DamAge	AST	0.75	0.29	2.63	9.11E‐03	*
AdaptAge	AST	0.37	0.34	1.08	0.28	
GrimAge V1	ALT	0.35	0.20	1.80	0.07	
GrimAge V2	ALT	0.49	0.21	2.38	0.02	·
CausAge	ALT	0.06	0.20	0.29	0.78	
DamAge	ALT	0.56	0.29	1.94	0.05	
AdaptAge	ALT	0.70	0.34	2.03	0.04	·
GrimAge V1	CRP	0.66	0.25	2.60	0.01	·
GrimAge V2	CRP	0.77	0.27	2.85	4.87E‐03	*
CausAge	CRP	−0.01	0.24	−0.04	0.97	
DamAge	CRP	−0.14	0.36	−0.39	0.69	
AdaptAge	CRP	−0.17	0.41	−0.42	0.68	
GrimAge V1	A1C	0.28	0.28	1.01	0.31	
GrimAge V2	A1C	0.24	0.30	0.81	0.42	
CausAge	A1C	0.06	0.27	0.21	0.83	
DamAge	A1C	−0.50	0.40	−1.25	0.21	
AdaptAge	A1C	0.08	0.50	0.15	0.88	

*Note*: Heavy drinking days are defined as greater than or equal to 4 drinks a day for females and greater than or equal to 5 drinks a day for males. *p*‐values were obtained through two‐sample *t*‐tests.

Abbreviations: A1C, hemoglobin A1C; ALT, alanine aminotransferase; AST, aspartate aminotransferase; AUD, alcohol use disorder; CRP, C‐reactive protein; GGT, gamma‐glutamyl transferase; V1, Version 1; V2, Version 2.

Significance codes are as follows: “***” when *p*‐value (*p*) < 0.0001, “**” when *p* < 0.001, “*” when *p* < 0.01, “·” when *p* < 0.05.

In the old (≥40 years old) subgroup, GrimAge V2 also maintained a bit more of a significant association with AUD (*β* = 3.13, *p* = 4.34E‐08) compared with GrimAge V1 (*β* = 2.73, *p* = 1.13E‐07) (Table 4). Associations with total drinks, average number of drinks per day, and number of heavy drinking days were more significant for GrimAge V2 (*p*s < 0.01) (Table 4). Significant associations with GGT, AST, ALT, and CRP reflect this pattern as well (Table 4). Altogether, GrimAge V2 was typically slightly more associated with alcohol consumption behaviors, LFEs, and CRP compared with GrimAge V1 (Table 4). For the causality‐enriched clocks, none of the causality‐enriched epigenetic clocks showed any significant associations with LFEs or the alternative clinical variables except AdaptAge with A1C (*β* = 1.44, *p* < 0.01) (Table 4).

**TABLE 4:** Associations of epigenetic clocks with alcohol consumption‐based biomarkers in the old cohort (≥40 years old).

Model	Alcohol variable	*β*	SE	*T*	*p*‐value	Significance
GrimAge V1	AUD status	2.73	0.50	5.43	1.13E‐07	***
GrimAge V2	AUD status	3.13	0.56	5.62	4.34E‐08	***
CausAge	AUD status	0.20	0.55	0.37	0.71	
DamAge	AUD status	0.18	0.74	0.24	0.81	
AdaptAge	AUD status	0.72	0.94	0.76	0.45	
GrimAge V1	Total number of drinks	0.65	0.22	2.99	3.00E‐03	*
GrimAge V2	Total number of drinks	0.76	0.24	3.15	1.81E‐03	*
CausAge	Total number of drinks	0.10	0.24	0.41	0.68	
DamAge	Total number of drinks	0.04	0.33	0.12	0.91	
AdaptAge	Total number of drinks	−0.32	0.41	−0.78	0.44	
GrimAge V1	Number of drinking days	0.08	0.27	0.30	0.76	
GrimAge V2	Number of drinking days	0.08	0.30	0.27	0.78	
CausAge	Number of drinking days	0.24	0.29	0.82	0.41	
DamAge	Number of drinking days	−0.47	0.39	−1.19	0.23	
AdaptAge	Number of drinking days	0.68	0.50	1.36	0.17	
GrimAge V1	Average number of drinks per day	0.75	0.22	3.35	9.25E‐04	**
GrimAge V2	Average number of drinks per day	0.84	0.25	3.40	7.65E‐04	**
CausAge	Average number of drinks per day	0.18	0.25	0.73	0.47	
DamAge	Average number of drinks per day	0.31	0.34	0.93	0.35	
AdaptAge	Average number of drinks per day	−0.60	0.42	−1.43	0.15	
GrimAge V1	Number of heavy drinking days	0.85	0.26	3.22	1.40E‐03	*
GrimAge V2	Number of heavy drinking days	0.95	0.29	3.24	1.31E‐03	*
CausAge	Number of heavy drinking days	0.15	0.29	0.53	0.60	
DamAge	Number of heavy drinking days	0.11	0.40	0.29	0.77	
AdaptAge	Number of heavy drinking days	0.02	0.50	0.04	0.97	
GrimAge V1	GGT	0.53	0.19	2.85	4.67E‐03	*
GrimAge V2	GGT	0.73	0.21	3.56	4.22E‐04	**
CausAge	GGT	0.38	0.20	1.85	0.07	
DamAge	GGT	0.70	0.28	2.54	0.01	·
AdaptAge	GGT	0.02	0.36	0.06	0.95	
GrimAge V1	AST	0.51	0.19	2.66	8.27E‐03	*
GrimAge V2	AST	0.73	0.21	3.45	6.46E‐04	**
CausAge	AST	0.15	0.21	0.70	0.48	
DamAge	AST	0.60	0.28	2.11	0.04	·
AdaptAge	AST	−0.55	0.36	−1.52	0.13	
GrimAge V1	ALT	0.40	0.20	2.03	0.04	·
GrimAge V2	ALT	0.59	0.22	2.71	7.19E‐03	*
CausAge	ALT	0.09	0.22	0.40	0.69	
DamAge	ALT	−0.19	0.29	−0.64	0.53	
AdaptAge	ALT	0.08	0.37	0.21	0.84	
GrimAge V1	CRP	0.62	0.20	3.02	2.78E‐03	*
GrimAge V2	CRP	0.77	0.23	3.39	8.11E‐04	**
CausAge	CRP	−0.24	0.23	−1.07	0.28	
DamAge	CRP	0.06	0.31	0.20	0.84	
AdaptAge	CRP	0.42	0.40	1.05	0.30	
GrimAge V1	A1C	0.51	0.25	2.06	0.04	·
GrimAge V2	A1C	0.55	0.28	1.97	0.05	
CausAge	A1C	0.39	0.27	1.43	0.15	
DamAge	A1C	−0.08	0.39	−0.21	0.84	
AdaptAge	A1C	1.44	0.54	2.70	7.75E‐03	*

*Note*: Heavy drinking days are defined as greater than or equal to 4 drinks a day for females and greater than or equal to 5 drinks a day for males. *p*‐values were obtained through two‐sample *t*‐tests.

Abbreviations: A1C, hemoglobin A1C; ALT, alanine aminotransferase; AST, aspartate aminotransferase; AUD, alcohol use disorder; CRP, C‐reactive protein; GGT, gamma‐glutamyl transferase; V1, Version 1; V2, Version 2.

Significance codes are as follows: “***” when *p*‐value (*p*) < 0.0001, “**” when *p* < 0.001, “*” when *p* < 0.01, “.” when *p* < 0.05.

In expanded analyses evaluating sex‐and race‐specific subgroups, we observed similar patterns of associations between alcohol consumption‐related metrics and EAA (Tables S2.1–S3.2), with the exception that African American participants typically showed weaker associations across alcohol consumption‐related phenotypes.

### Propensity score analysis for retrospective matching

The propensity score analysis with the optimal matching approach selected 243 AUD patients and 243 HC individuals (1:1 matching), which left 129 AUD patients unmatched (Chen et al., 2022). The distribution of propensity scores in the two groups before and after matching suggested that the matched group was well‐balanced. Compared with the results of the total sample (Table 2), the results of the matched sample showed similar patterns of association with AUD status (with smaller effect sizes) and the alternate clinical biomarkers (i.e., CRP with smaller effect sizes). However, the results revealed dissimilar patterns of association with alcohol consumption behaviors, differing in magnitude (i.e., stronger associations of GrimAge V1 with total number of drinks, number of drinking days, average number of drinks per day) and LFEs (significant association of GrimAge V2 with GGT only) (Table S4). In a similar manner as the total sample analysis, the causality‐enriched clocks generally did not show any statistically significant associations with alcohol‐related metrics, with the only exception being DamAge and CausAge with only GGT (Table S4).

## DISCUSSION

In this study, we used several of the newest epigenetic clock models (GrimAge Version 1, GrimAge Version 2, CausAge, DamAge, and AdaptAge) to thoroughly examine the impact of AUD and alcohol consumption‐related phenotypes on biological aging. We found that biological age, as signified in the GrimAge V2 model, experiences greater EAA in the AUD group as compared to the HC group (*β* = 2.43, *p* = 1.50E‐09) (Table 2). This demonstrates slightly better performance in comparison with GrimAge V1 (*β* = 2.01, *p* = 3.70E‐08) (Table 2). This dynamic is consistent across both the old and young age subgroups as well as sex‐specific and race‐specific subgroups, further validating the improved power of GrimAge V2 in the AUD context. However, with the causality‐enriched epigenetic clocks, there were no significant associations between the AUD group and the CausAge, DamAge, and AdaptAge models (*p*s > 0.01) (Table 2).

We witnessed significant associations between EAA driven by GrimAge V1 and GrimAge V2 and alcohol consumption behaviors, as characterized by the total number of drinks in the past 90 days, average number of drinks per day, and number of heavy drinking days (*p*s < 0.0001) (Table 2). GrimAge V2's stronger association patterns were mimicked in the old subgroup, but not quite as much in the young subgroup. Differences in alcohol consumption due to societal norms and habits in contrasting age demographics may help to explain this (Delker et al., 2016).

With extended alcohol consumption biometric analysis, we found similar significant associations between both versions of GrimAge and elevated LFEs, especially with GGT and AST. Compared with GrimAge V1, GrimAge V2 upheld somewhat more significant associations with LFEs across the total sample, as well as the young and old cohorts, amidst the lack of a statistically significant difference in effect sizes between GrimAge V1 and V2 (*p*s > 0.05, Table S1). Comparing CausAge, DamAge, and AdaptAge, DamAge typically maintained significant associations with GGT and AST, while AdaptAge contradictorily showed significant associations with A1C, particularly in the old cohort. Such findings validate what has been discovered in prior studies that have demonstrated how harsher AUD‐related phenotypes are linked to acceleration in the biological aging process (Luo et al., 2020; Robinson et al., 2020). It is important to note, however, that CausAge's predictive power may have been skewed due to the chunk of relevant CpGs that were not available in the genetic dataset.

### Epigenetic clock evolution

In this study, we formulated more comprehensive comparisons between newly reported epigenetic clocks and well‐characterized alcohol‐related phenotypes. Previously, our studies compared associations of EAA with AUD and its associated endophenotypes, focusing on the first and second‐generation epigenetic clocks (Jung et al., 2023). Interestingly, only EAA from PhenoAge (defined as a methylation‐based prediction of composite phenotypic age) and GrimAge V1 were associated with severe alcohol‐related phenotypes, even though PhenoAge and GrimAge V1 lack overlap in the types of biomarkers used (Levine et al., 2018; Lu et al., 2019). The two clocks were shown to better differentiate morbidity and mortality risk among individuals of the same chronological age than the first‐generation epigenetic clocks. Furthermore, in contrast with GrimAge V1, both PhenoAge and GrimAge V2 integrate CRP levels; however, PhenoAge employs serum glucose levels while GrimAge V2 applies hemoglobin A1C levels (Levine et al., 2018; Lu et al., 2019, 2022).

In terms of predictive power, GrimAge V1 outperforms PhenoAge in regard to its extent of associations with both mortality and morbidity parameters, including time‐to‐death, time‐to‐cancer, time‐to‐CHD (coronary heart disease), age at menopause, and various comorbidities (Lu et al., 2019). Moreover, GrimAge V2 surpasses GrimAge V1 in predicting time‐to‐death resulting from all‐cause mortality, time‐to‐CHD, time‐to‐CHF (congestive heart failure), type 2 diabetes, hypertension, disease‐free status, physical functioning level, and again various comorbidities (amongst others) (Lu et al., 2022). Although a statistical test did not confirm a significant difference in effect sizes between GrimAge V1 and V2, we observed that GrimAge V2 generally outperformed GrimAge V1 across most alcohol behaviors as well as LFT measures. This was evident in both the magnitude of the effect sizes and their *p*‐values. Our findings align with previous research indicating that GrimAge V2 shows stronger associations with age‐related phenotypes compared with GrimAge V1. This improvement may be attributed to the incorporation of DNAm logCRP and DNAm logA1C, which are linked to numerous age‐related conditions (Lu et al., 2022) as well as alcohol‐associated behaviors.

Out of the three fourth‐generation clocks, DamAge features the higher degree of significance in positive association with mortality, with comparable efficacy to PhenoAge (Ying et al., 2024). Meanwhile, AdaptAge maintains a significant negative association with mortality (Ying et al., 2024). Looking at some contexts of clinical validation, with pluripotent stem cell reprogramming, DamAge and AdaptAge sustain clinically substantial associations, with DamAge showcasing the most substantial association out of the previously ratified clock models (Ying et al., 2024). Despite both DamAge and AdaptAge demonstrating significant associations (DamAge—strong positive associations, AdaptAge—strong negative associations) with several age‐related diseases such as hypertension, cancer, and Hutchinson–Gilford progeria (HGP), our findings showed that these clocks were not associated with alcohol‐induced phenotypes and AUD.

### Clinical implications

By additionally incorporating DNAm‐based estimators of high sensitivity‐CRP, a widely used biomarker of inflammation, and hemoglobin A1C, a common biomarker for assessing blood glucose levels, GrimAge V2 showed a more comprehensive and sensitive prediction of human mortality and morbidity than GrimAge V1. To start, both plasma proteins are involved in alcohol metabolism pathways (Bell et al., 2000; Lindtner et al., 2013; Wang et al., 2010). For example, AUD corresponds to short‐and long‐term alcohol intake that disrupts the management of glucose and consequently A1C levels (Wiss, 2019). However, the dynamics between alcohol and blood sugar concentration appear to fluctuate depending on drinking intensity and duration (Athyros et al., 2007; Facchini et al., 1994). Similar to A1C, CRP indicates a relationship between chronic alcohol use and inflammatory pathways and also shows associations with age‐related health conditions (Rosen et al., 2018; State, 2021). Our studies may suggest incorporation of the A1C and CRP biomarkers in future epigenetic clock models to potentially provide a better understanding of the effects of alcohol on advanced age acceleration (Bell et al., 2000; Caruana et al., 2015). However, further studies are necessary to see whether A1C and CRP directly contribute to the mechanisms of alcohol‐related metabolism.

More broadly, the GrimAge models outperform the fourth‐generation clocks to a considerable degree. Given the differences in construction and CpG sites selected, the GrimAge foundation appears to incorporate CpGs with more predictive power. One potential candidate would be cg05575921, which is found on the aryl‐hydrocarbon receptor (AHRR) gene and has been linked to smoking (Bravo‐Gutierrez et al., 2021). Smoking pack‐years was included as a clinical biomarker in both GrimAge models, to no surprise given the well‐established associations of smoking with mortality (Bravo‐Gutierrez et al., 2021). However, smoking has also been found to interact with alcohol consumption (Verplaetse & McKee, 2017). Studies have shown that alcohol can elevate the desire to smoke at both smaller and larger quantities; meanwhile, nicotine in tobacco can dilute the soporific effects of alcohol, thus suggesting a complex dynamic between the two substances that may be better encapsulated by the GrimAge model (Verplaetse & McKee, 2017). Furthermore, the GrimAge clocks were designed to predict time to death (due to all‐cause mortality) using DNAm‐based age‐related plasma protein biomarkers and DNAm‐smoking packs per year, while the causality‐enriched epigenetic clocks were developed to predict chronological age using CpGs identified by an epigenome‐wide Mendelian randomization (EWMR) approach and reliance on age‐associated differential methylation. Many studies have shown that the first‐generation clocks predicting chronological age tend to underperform in comparison to the second‐generation clocks in regard to associations with various health conditions (Levine et al., 2018; Robinson et al., 2020).

The fourth‐generation clocks (CausAge, DamAge, and AdaptAge) were founded upon CpG sites deemed causal to aging as represented by lifespan, extreme longevity, health span, frailty index, self‐rated health, aging‐GIP1, socioeconomic traits‐adjusted aging‐GIP1, and healthy aging (Ying et al., 2024). To clarify, GIP1 (genetically independent phenotype, principal component 1) is derived from health span, mother's lifespan, father's lifespan, remarkable longevity, index of frailty, and self‐assessed health (Timmers et al., 2022). Despite the secondary performance of these models in comparison with the GrimAge clocks, they may be useful in whittling down the list of significant candidates regarding health outcome‐associated epigenetic influences on biological age.

### Strengths

Our study has several strengths, including a well‐characterized sample and enrichment of alcohol‐related clinical phenotypes. Our sample consists of individuals from European and African American ancestries. In the AUD group, 47.58% identified as African Americans and 51.34% as European Americans (Table 1). The over‐representation of African Americans in our sample extends our findings' generalizability to ancestral groups that are not often represented in research studies. In addition, the enrichment of alcohol‐related clinical phenotypes provides opportunities for conducting various endophenotypic analyses, including disease severity analyses.

### Limitations

While our investigation had adequate power to detect the association of EAA with alcohol consumption‐related behaviors, we cannot confirm causal relationships between these variables. Specifically, we cannot definitively demonstrate whether AUD is a risk factor for accelerated epigenetic aging or a consequence of chronic alcohol use using the epigenetic clock models. Our study involves a cross‐sectional analysis, where several variables are considered at a singular point in time. As such, the study is unable to analyze two separate time points for the same individual, so there is a lack of longitudinal character that ultimately prevents any solidified conclusions in regard to causality prediction by the epigenetic clock models (Caruana et al., 2015). To address this limitation of our cross‐sectional study, future studies could incorporate the collection of methylation data at multiple time points to investigate the effect of long‐term alcohol use on aging processes. In addition, there may be potential bias due to the self‐reporting nature of the questionnaires filled out by participants in the studies covered by the data set (Jung et al., 2023). Furthermore, our study was limited in collecting data on other environmental/lifestyle factors (e.g., exercise and diet) that may influence DNA methylation levels, and therefore, these factors were not included in our analyses. Nevertheless, we did account for age, sex, race, BMI, and smoking status, which all have been shown to be significantly associated with epigenetic aging (Hannum et al., 2013; Horvath, 2013; Levine et al., 2018; Lu et al., 2019, 2022). Although our study examined methylation in whole blood only and controlled for blood cell type composition, expansion to tissues and cell methylation samples may be necessary to validate alcohol‐related aging. Furthermore, the differences in chip compatibilities with the causality‐enriched clocks, where CausAge was missing a considerable number of probes, make comparison of performance a challenge. As such, the CausAge associations may have been much more skewed (Ying et al., 2024).

## CONCLUSIONS

Our research study highlights the differences in associative power between DNAm GrimAge V1 and V2, as well as those among three causality‐enriched epigenetic clocks (CausAge, DamAge, and AdaptAge) in respect to AUD and various well‐characterized alcohol consumption‐related endophenotypes. By using a more phenotypically comprehensive and demographically balanced sample, our findings can be expanded to broader populations, which sparks potential in the development of diagnostic and therapeutic treatments. Improved performance following incorporation of A1C and CRP in the GrimAge V2 model elicits further investigation into the alcohol‐based interactions involving these plasma proteins.

## FUNDING INFORMATION

This work was supported by the National Institutes of Health (NIH) intramural funding (ZIA‐AA000242 to F.W.L.) as part of the DICBR of the NIAAA. The NIAAA Natural History protocol was supported by the NIH intramural funding (ZIA‐AA000130 to N.D.) as part of the DICBR of the NIAAA.

## CONFLICT OF INTEREST STATEMENT

The authors declare no competing interests.

## Supporting information


Appendix S1

## Data Availability

The data that support the findings of this study are available from the corresponding author upon reasonable request.
