# Supplementary material for: Alcohol and aging: Next‐generation epigenetic clocks predict biological age acceleration in individuals with alcohol use disorder
Source: Alcohol Clin Exp Res (Hoboken). 2025 Mar 28;49(4):829–42. doi: 10.1111/acer.70020 (PMC12012873; doi:10.1111/acer.70020)
Supplement: Supplementary file 1 — Appendix S1 [file ACER-49-829-s001.docx]

SUPPLEMENTAL TABLES

**The theoretical formula of the effect size comparison between DNAm GrimAge Version 1 (V1) and Version 2 (V2)**

The formal test of difference between the b_1_ (effect size) of DNAm GrimAge V1 and b_2_ (effect size) of DNAm GrimAge V2 in the same sample is presented below.

Let$y_{1}$ be the age-adjusted GrimAge V1 and $y_{2}$ be the age-adjusted GrimAge V2. $X_{1}$ is the alcohol consumption-related variable of interest, such as GGT:

$y_{1}=b_{1}X_{1}+e_{1}$ (1)

$y_{2}=b_{2}X_{1}+e_{2}$ (2)

$$y_{1}-y_{2}={(b}_{1}-b_{2}{)X}_{1}+covariates+ ({e_{1}-e}_{2})$$

We then conducted statistical analysis with the difference between the two age-adjusted GrimAge clocks as the dependent variable, the alcohol consumption-related variable of interest (e.g. GGT) as the independent variable, and with adjustment for covariates.

$$T=\frac{b_{1}-b_{2}}{SE(b_{1}-b_{2})}$$

The P-values of the T-statistics were estimated and are presented in Supplementary Table 1.

**Supplementary Table 1. Statistical test of difference in effect sizes between DNAm GrimAge Version 1 (V1) and Version 2 (V2) with alcohol consumption-based biomarkers in the total sample, young cohort (< 40 years old), and old cohort (>= 40 years old).**

| Demographic | Alcohol Variable | β_V1_ | β_V2_ | T | P-value | Significance |
| --- | --- | --- | --- | --- | --- | --- |
|  |  |  |  |  |  |  |
| Total Sample | AUD Status | 2.01 | 2.43 | -0.78 | 0.44 |  |
|  | Total Number of Drinks | 0.82 | 0.95 | -0.50 | 0.62 |  |
|  | Number of Drinking Days | 0.23 | 0.26 | -0.10 | 0.92 |  |
|  | Average Number of Drinks Per Day | 0.78 | 0.87 | -0.34 | 0.73 |  |
|  | Number of Heavy Drinking Days | 1.07 | 1.17 | -0.29 | 0.77 |  |
|  | GGT | 0.55 | 0.76 | -1.03 | 0.30 |  |
|  | AST | 0.47 | 0.67 | -0.96 | 0.34 |  |
|  | ALT | 0.36 | 0.53 | -0.78 | 0.43 |  |
|  | CRP | 0.64 | 0.77 | -0.50 | 0.62 |  |
|  | A1C | 0.41 | 0.40 | 0.05 | 0.96 |  |
|  |  |  |  |  |  |  |
| Young Cohort | AUD Status | 1.59 | 1.99 | -0.55 | 0.58 |  |
|  | Total Number of Drinks | 1.09 | 1.18 | -0.24 | 0.81 |  |
|  | Number of Drinking Days | 0.32 | 0.35 | -0.07 | 0.95 |  |
|  | Average Number of Drinks Per Day | 0.83 | 0.85 | -0.05 | 0.96 |  |
|  | Number of Heavy Drinking Days | 1.33 | 1.37 | -0.07 | 0.94 |  |
|  | GGT | 0.36 | 0.58 | -0.78 | 0.44 |  |
|  | AST | 0.27 | 0.42 | -0.53 | 0.60 |  |
|  | ALT | 0.35 | 0.49 | -0.49 | 0.63 |  |
|  | CRP | 0.66 | 0.77 | -0.29 | 0.77 |  |
|  | A1C | 0.28 | 0.24 | 0.10 | 0.92 |  |
|  |  |  |  |  |  |  |
| Old Cohort | AUD Status | 2.73 | 3.13 | -0.54 | 0.59 |  |
|  | Total Number of Drinks | 0.65 | 0.76 | -0.33 | 0.74 |  |
|  | Number of Drinking Days | 0.08 | 0.08 | -4.19E-04 | > 0.99 |  |
|  | Average Number of Drinks Per Day | 0.75 | 0.84 | -0.28 | 0.78 |  |
|  | Number of Heavy Drinking Days | 0.85 | 0.95 | -0.25 | 0.80 |  |
|  | GGT | 0.53 | 0.73 | -0.73 | 0.47 |  |
|  | AST | 0.51 | 0.73 | -0.77 | 0.44 |  |
|  | ALT | 0.40 | 0.59 | -0.64 | 0.52 |  |
|  | CRP | 0.62 | 0.77 | -0.50 | 0.62 |  |
|  | A1C | 0.51 | 0.55 | -0.11 | 0.91 |  |
| Note: AUD = alcohol use disorder; GGT = gamma-glutamyl transferase; AST = aspartate aminotransferase; ALT = alanine aminotransferase; CRP = C-reactive protein; A1C = hemoglobin A1C. Heavy drinking days are defined as greater than or equal to 4 drinks a day for females and greater than or equal to 5 drinks a day for males. Significance codes are as follows: ‘***’ when P-value (P) < 0.0001, ‘**’ when P < 0.001, ‘*’ when P < 0.01, ‘.’ when P < 0.05. P-values were obtained through two sample t-tests. | | | | | | |

**Supplementary Table 2.1. Associations of epigenetic clocks with alcohol consumption-based biomarkers in the male cohort.**

| Model | Alcohol Variable | β | SE | T | P-value | Significance |
| --- | --- | --- | --- | --- | --- | --- |
|  |  |  |  |  |  |  |
| GrimAge V1 | AUD Status | 1.75 | 0.47 | 3.73 | 2.27E-04 | ** |
| GrimAge V2 | AUD Status | 2.11 | 0.51 | 4.13 | 4.53E-05 | *** |
| CausAge | AUD Status | 0.50 | 0.51 | 0.99 | 0.32 |  |
| DamAge | AUD Status | 0.29 | 0.71 | 0.41 | 0.68 |  |
| AdaptAge | AUD Status | 0.84 | 0.86 | 0.98 | 0.33 |  |
|  |  |  |  |  |  |  |
| GrimAge V1 | Total Number of Drinks | 0.75 | 0.22 | 3.35 | 9.14E-04 | ** |
| GrimAge V2 | Total Number of Drinks | 0.84 | 0.24 | 3.45 | 6.41E-04 | ** |
| CausAge | Total Number of Drinks | 0.28 | 0.24 | 1.14 | 0.26 |  |
| DamAge | Total Number of Drinks | 0.69 | 0.34 | 2.03 | 0.04 | . |
| AdaptAge | Total Number of Drinks | -0.17 | 0.41 | -0.40 | 0.69 |  |
|  |  |  |  |  |  |  |
| GrimAge V1 | Number of Drinking Days | 0.11 | 0.28 | 0.39 | 0.70 |  |
| GrimAge V2 | Number of Drinking Days | 0.15 | 0.31 | 0.47 | 0.64 |  |
| CausAge | Number of Drinking Days | -0.11 | 0.31 | -0.37 | 0.72 |  |
| DamAge | Number of Drinking Days | -0.23 | 0.43 | -0.54 | 0.59 |  |
| AdaptAge | Number of Drinking Days | 0.35 | 0.52 | 0.67 | 0.50 |  |
|  |  |  |  |  |  |  |
| GrimAge V1 | Average Number of Drinks Per Day | 0.74 | 0.23 | 3.27 | 1.21E-03 | * |
| GrimAge V2 | Average Number of Drinks Per Day | 0.79 | 0.25 | 3.17 | 1.68E-03 | * |
| CausAge | Average Number of Drinks Per Day | 0.33 | 0.24 | 1.34 | 0.18 |  |
| DamAge | Average Number of Drinks Per Day | 0.78 | 0.34 | 2.29 | 0.02 | . |
| AdaptAge | Average Number of Drinks Per Day | -0.29 | 0.42 | -0.69 | 0.49 |  |
|  |  |  |  |  |  |  |
| GrimAge V1 | Number of Heavy Drinking Days | 1.00 | 0.28 | 3.56 | 4.28E-04 | ** |
| GrimAge V2 | Number of Heavy Drinking Days | 1.01 | 0.31 | 3.29 | 1.12E-03 | * |
| CausAge | Number of Heavy Drinking Days | 0.14 | 0.31 | 0.45 | 0.66 |  |
| DamAge | Number of Heavy Drinking Days | 0.66 | 0.43 | 1.54 | 0.13 |  |
| AdaptAge | Number of Heavy Drinking Days | -0.01 | 0.52 | -0.02 | 0.98 |  |
|  |  |  |  |  |  |  |
| GrimAge V1 | GGT | 0.43 | 0.19 | 2.31 | 0.02 | . |
| GrimAge V2 | GGT | 0.65 | 0.20 | 3.22 | 1.39E-03 | * |
| CausAge | GGT | 0.50 | 0.20 | 2.47 | 0.01 | . |
| DamAge | GGT | 1.06 | 0.28 | 3.82 | 1.60E-04 | ** |
| AdaptAge | GGT | -0.33 | 0.34 | -0.94 | 0.35 |  |
|  |  |  |  |  |  |  |
| GrimAge V1 | AST | 0.51 | 0.19 | 2.70 | 7.31E-03 | * |
| GrimAge V2 | AST | 0.68 | 0.20 | 3.38 | 8.01E-04 | ** |
| CausAge | AST | 0.49 | 0.20 | 2.44 | 0.02 | . |
| DamAge | AST | 1.34 | 0.28 | 4.87 | 1.73E-06 | *** |
| AdaptAge | AST | -0.35 | 0.35 | -1.00 | 0.32 |  |
|  |  |  |  |  |  |  |
| GrimAge V1 | ALT | 0.44 | 0.19 | 2.31 | 0.02 | . |
| GrimAge V2 | ALT | 0.59 | 0.20 | 2.86 | 4.46E-03 | * |
| CausAge | ALT | 0.27 | 0.20 | 1.33 | 0.18 |  |
| DamAge | ALT | 0.54 | 0.29 | 1.91 | 0.06 |  |
| AdaptAge | ALT | 0.25 | 0.35 | 0.71 | 0.48 |  |
|  |  |  |  |  |  |  |
| GrimAge V1 | CRP | 0.47 | 0.23 | 2.05 | 0.04 | . |
| GrimAge V2 | CRP | 0.54 | 0.25 | 2.17 | 0.03 | . |
| CausAge | CRP | -0.12 | 0.24 | -0.52 | 0.60 |  |
| DamAge | CRP | -0.03 | 0.33 | -0.08 | 0.93 |  |
| AdaptAge | CRP | 0.37 | 0.40 | 0.95 | 0.35 |  |
|  |  |  |  |  |  |  |
| GrimAge V1 | A1C | -0.05 | 0.27 | -0.19 | 0.85 |  |
| GrimAge V2 | A1C | -0.15 | 0.29 | -0.50 | 0.62 |  |
| CausAge | A1C | 0.49 | 0.27 | 1.80 | 0.07 |  |
| DamAge | A1C | -0.55 | 0.39 | -1.38 | 0.17 |  |
| AdaptAge | A1C | 0.85 | 0.52 | 1.63 | 0.10 |  |
| Note: AUD = alcohol use disorder; GGT = gamma-glutamyl transferase; AST = aspartate aminotransferase; ALT = alanine aminotransferase; CRP = C-reactive protein; A1C = hemoglobin A1C; V1 = Version 1; V2 = Version 2. Heavy drinking days are defined as greater than or equal to 4 drinks a day for females and greater than or equal to 5 drinks a day for males. Significance codes are as follows: ‘***’ when P-value (P) < 0.0001, ‘**’ when P < 0.001, ‘*’ when P < 0.01, ‘.’ when P < 0.05. P-values were obtained through two sample t-tests. | | | | | | |

**Supplementary Table 2.2. Associations of epigenetic clocks with alcohol consumption-based biomarkers in the female cohort.**

| Model | Alcohol Variable | β | SE | T | P-value | Significance |
| --- | --- | --- | --- | --- | --- | --- |
|  |  |  |  |  |  |  |
| GrimAge V1 | AUD Status | 2.26 | 0.59 | 3.83 | 1.60E-04 | ** |
| GrimAge V2 | AUD Status | 2.87 | 0.65 | 4.41 | 1.50E-05 | *** |
| CausAge | AUD Status | 0.15 | 0.57 | 0.25 | 0.80 |  |
| DamAge | AUD Status | 1.13 | 0.80 | 1.41 | 0.16 |  |
| AdaptAge | AUD Status | 0.85 | 0.99 | 0.86 | 0.39 |  |
|  |  |  |  |  |  |  |
| GrimAge V1 | Total Number of Drinks | 1.13 | 0.29 | 3.95 | 1.01E-04 | ** |
| GrimAge V2 | Total Number of Drinks | 1.30 | 0.31 | 4.15 | 4.47E-05 | *** |
| CausAge | Total Number of Drinks | 0.42 | 0.28 | 1.48 | 0.14 |  |
| DamAge | Total Number of Drinks | 0.13 | 0.40 | 0.31 | 0.75 |  |
| AdaptAge | Total Number of Drinks | 0.14 | 0.50 | 0.28 | 0.78 |  |
|  |  |  |  |  |  |  |
| GrimAge V1 | Number of Drinking Days | 0.38 | 0.34 | 1.10 | 0.27 |  |
| GrimAge V2 | Number of Drinking Days | 0.45 | 0.38 | 1.18 | 0.24 |  |
| CausAge | Number of Drinking Days | 0.81 | 0.33 | 2.44 | 0.02 | . |
| DamAge | Number of Drinking Days | 0.43 | 0.47 | 0.92 | 0.36 |  |
| AdaptAge | Number of Drinking Days | 0.97 | 0.58 | 1.67 | 0.10 |  |
|  |  |  |  |  |  |  |
| GrimAge V1 | Average Number of Drinks Per Day | 0.95 | 0.30 | 3.10 | 2.15E-03 | * |
| GrimAge V2 | Average Number of Drinks Per Day | 1.07 | 0.34 | 3.19 | 1.64E-03 | * |
| CausAge | Average Number of Drinks Per Day | 0.28 | 0.30 | 0.91 | 0.36 |  |
| DamAge | Average Number of Drinks Per Day | 0.04 | 0.43 | 0.10 | 0.92 |  |
| AdaptAge | Average Number of Drinks Per Day | -0.13 | 0.52 | -0.25 | 0.80 |  |
|  |  |  |  |  |  |  |
| GrimAge V1 | Number of Heavy Drinking Days | 1.17 | 0.38 | 3.09 | 2.25E-03 | * |
| GrimAge V2 | Number of Heavy Drinking Days | 1.40 | 0.42 | 3.36 | 8.89E-04 | ** |
| CausAge | Number of Heavy Drinking Days | 0.48 | 0.37 | 1.28 | 0.20 |  |
| DamAge | Number of Heavy Drinking Days | 0.20 | 0.53 | 0.38 | 0.71 |  |
| AdaptAge | Number of Heavy Drinking Days | 0.24 | 0.65 | 0.37 | 0.71 |  |
|  |  |  |  |  |  |  |
| GrimAge V1 | GGT | 0.71 | 0.21 | 3.39 | 8.15E-04 | ** |
| GrimAge V2 | GGT | 0.91 | 0.23 | 4.00 | 8.29E-05 | *** |
| CausAge | GGT | 0.32 | 0.21 | 1.57 | 0.12 |  |
| DamAge | GGT | 0.54 | 0.29 | 1.87 | 0.06 |  |
| AdaptAge | GGT | 0.75 | 0.36 | 2.10 | 0.04 | . |
|  |  |  |  |  |  |  |
| GrimAge V1 | AST | 0.43 | 0.22 | 2.00 | 0.05 | . |
| GrimAge V2 | AST | 0.68 | 0.24 | 2.84 | 4.84E-03 | * |
| CausAge | AST | -0.02 | 0.21 | -0.10 | 0.92 |  |
| DamAge | AST | 0.08 | 0.30 | 0.28 | 0.78 |  |
| AdaptAge | AST | 0.15 | 0.37 | 0.40 | 0.69 |  |
|  |  |  |  |  |  |  |
| GrimAge V1 | ALT | 0.25 | 0.22 | 1.14 | 0.26 |  |
| GrimAge V2 | ALT | 0.43 | 0.24 | 1.78 | 0.08 |  |
| CausAge | ALT | -0.08 | 0.21 | -0.38 | 0.70 |  |
| DamAge | ALT | -0.19 | 0.30 | -0.63 | 0.53 |  |
| AdaptAge | ALT | 0.53 | 0.37 | 1.43 | 0.15 |  |
|  |  |  |  |  |  |  |
| GrimAge V1 | CRP | 1.14 | 0.26 | 4.44 | 1.53E-05 | *** |
| GrimAge V2 | CRP | 1.43 | 0.28 | 5.01 | 1.31E-06 | *** |
| CausAge | CRP | -0.11 | 0.26 | -0.43 | 0.67 |  |
| DamAge | CRP | 0.29 | 0.38 | 0.77 | 0.44 |  |
| AdaptAge | CRP | 0.38 | 0.45 | 0.84 | 0.40 |  |
|  |  |  |  |  |  |  |
| GrimAge V1 | A1C | 0.99 | 0.28 | 3.50 | 6.42E-04 | ** |
| GrimAge V2 | A1C | 1.09 | 0.32 | 3.41 | 8.51E-04 | ** |
| CausAge | A1C | -0.14 | 0.31 | -0.47 | 0.64 |  |
| DamAge | A1C | -0.13 | 0.45 | -0.30 | 0.77 |  |
| AdaptAge | A1C | 0.96 | 0.56 | 1.71 | 0.09 |  |
| Note: AUD = alcohol use disorder; GGT = gamma-glutamyl transferase; AST = aspartate aminotransferase; ALT = alanine aminotransferase; CRP = C-reactive protein; A1C = hemoglobin A1C; V1 = Version 1; V2 = Version 2. Heavy drinking days are defined as greater than or equal to 4 drinks a day for females and greater than or equal to 5 drinks a day for males. Significance codes are as follows: ‘***’ when P-value (P) < 0.0001, ‘**’ when P < 0.001, ‘*’ when P < 0.01, ‘.’ when P < 0.05. P-values were obtained through two sample t-tests. | | | | | | |

**Supplementary Table 3.1. Associations of epigenetic clocks with alcohol consumption-based biomarkers in the European American cohort.**

| Model | Alcohol Variable | β | SE | T | P-value | Significance |
| --- | --- | --- | --- | --- | --- | --- |
|  |  |  |  |  |  |  |
| GrimAge V1 | AUD Status | 2.78 | 0.51 | 5.46 | 9.51E-08 | *** |
| GrimAge V2 | AUD Status | 3.25 | 0.57 | 5.72 | 2.45E-08 | *** |
| CausAge | AUD Status | 1.08 | 0.53 | 2.03 | 0.04 | . |
| DamAge | AUD Status | 1.71 | 0.76 | 2.25 | 0.03 | . |
| AdaptAge | AUD Status | 0.26 | 0.94 | 0.27 | 0.78 |  |
|  |  |  |  |  |  |  |
| GrimAge V1 | Total Number of Drinks | 0.97 | 0.23 | 4.13 | 4.72E-05 | *** |
| GrimAge V2 | Total Number of Drinks | 1.08 | 0.26 | 4.14 | 4.56E-05 | *** |
| CausAge | Total Number of Drinks | 0.34 | 0.25 | 1.34 | 0.18 |  |
| DamAge | Total Number of Drinks | 0.66 | 0.36 | 1.86 | 0.06 |  |
| AdaptAge | Total Number of Drinks | 0.19 | 0.44 | 0.43 | 0.67 |  |
|  |  |  |  |  |  |  |
| GrimAge V1 | Number of Drinking Days | 0.17 | 0.26 | 0.65 | 0.52 |  |
| GrimAge V2 | Number of Drinking Days | 0.17 | 0.29 | 0.60 | 0.55 |  |
| CausAge | Number of Drinking Days | 0.35 | 0.27 | 1.27 | 0.20 |  |
| DamAge | Number of Drinking Days | 0.30 | 0.39 | 0.77 | 0.44 |  |
| AdaptAge | Number of Drinking Days | 0.87 | 0.48 | 1.83 | 0.07 |  |
|  |  |  |  |  |  |  |
| GrimAge V1 | Average Number of Drinks Per Day | 1.00 | 0.25 | 4.00 | 7.90E-05 | *** |
| GrimAge V2 | Average Number of Drinks Per Day | 1.08 | 0.28 | 3.87 | 1.35E-04 | ** |
| CausAge | Average Number of Drinks Per Day | 0.38 | 0.27 | 1.42 | 0.16 |  |
| DamAge | Average Number of Drinks Per Day | 0.89 | 0.38 | 2.35 | 0.02 | . |
| AdaptAge | Average Number of Drinks Per Day | -0.20 | 0.48 | -0.42 | 0.68 |  |
|  |  |  |  |  |  |  |
| GrimAge V1 | Number of Heavy Drinking Days | 1.03 | 0.32 | 3.25 | 1.28E-03 | * |
| GrimAge V2 | Number of Heavy Drinking Days | 1.16 | 0.35 | 3.31 | 1.04E-03 | * |
| CausAge | Number of Heavy Drinking Days | 0.25 | 0.33 | 0.75 | 0.46 |  |
| DamAge | Number of Heavy Drinking Days | 0.57 | 0.48 | 1.19 | 0.24 |  |
| AdaptAge | Number of Heavy Drinking Days | 0.59 | 0.59 | 1.00 | 0.32 |  |
|  |  |  |  |  |  |  |
| GrimAge V1 | GGT | 0.56 | 0.18 | 3.09 | 2.16E-03 | * |
| GrimAge V2 | GGT | 0.80 | 0.20 | 4.02 | 7.30E-05 | *** |
| CausAge | GGT | 0.49 | 0.19 | 2.60 | 9.67E-03 | * |
| DamAge | GGT | 0.98 | 0.27 | 3.66 | 2.95E-04 | ** |
| AdaptAge | GGT | 0.44 | 0.34 | 1.29 | 0.20 |  |
|  |  |  |  |  |  |  |
| GrimAge V1 | AST | 0.47 | 0.18 | 2.57 | 0.01 | . |
| GrimAge V2 | AST | 0.73 | 0.20 | 3.57 | 4.16E-04 | ** |
| CausAge | AST | 0.34 | 0.19 | 1.75 | 0.08 |  |
| DamAge | AST | 1.00 | 0.27 | 3.65 | 3.08E-04 | ** |
| AdaptAge | AST | 0.05 | 0.34 | 0.15 | 0.88 |  |
|  |  |  |  |  |  |  |
| GrimAge V1 | ALT | 0.40 | 0.19 | 2.12 | 0.04 | . |
| GrimAge V2 | ALT | 0.62 | 0.21 | 2.99 | 3.05E-03 | * |
| CausAge | ALT | 0.19 | 0.20 | 0.98 | 0.33 |  |
| DamAge | ALT | 0.46 | 0.28 | 1.64 | 0.10 |  |
| AdaptAge | ALT | 0.66 | 0.35 | 1.92 | 0.06 |  |
|  |  |  |  |  |  |  |
| GrimAge V1 | CRP | 0.72 | 0.22 | 3.33 | 1.00E-03 | * |
| GrimAge V2 | CRP | 0.83 | 0.24 | 3.43 | 7.27E-04 | ** |
| CausAge | CRP | -0.12 | 0.22 | -0.55 | 0.58 |  |
| DamAge | CRP | 0.02 | 0.32 | 0.07 | 0.94 |  |
| AdaptAge | CRP | 0.44 | 0.39 | 1.15 | 0.25 |  |
|  |  |  |  |  |  |  |
| GrimAge V1 | A1C | 0.30 | 0.26 | 1.14 | 0.26 |  |
| GrimAge V2 | A1C | 0.03 | 0.29 | 0.10 | 0.92 |  |
| CausAge | A1C | 0.96 | 0.26 | 3.68 | 3.15E-04 | ** |
| DamAge | A1C | 0.22 | 0.40 | 0.56 | 0.58 |  |
| AdaptAge | A1C | 1.41 | 0.54 | 2.60 | 0.01 | . |
| Note: AUD = alcohol use disorder; GGT = gamma-glutamyl transferase; AST = aspartate aminotransferase; ALT = alanine aminotransferase; CRP = C-reactive protein; A1C = hemoglobin A1C; V1 = Version 1; V2 = Version 2. Heavy drinking days are defined as greater than or equal to 4 drinks a day for females and greater than or equal to 5 drinks a day for males. Significance codes are as follows: ‘***’ when P-value (P) < 0.0001, ‘**’ when P < 0.001, ‘*’ when P < 0.01, ‘.’ when P < 0.05. P-values were obtained through two sample t-tests. | | | | | | |

**Supplementary Table 3.2. Associations of epigenetic clocks with alcohol consumption-based biomarkers in the African American cohort.**

| Model | Alcohol Variable | β | SE | T | P-value | Significance |
| --- | --- | --- | --- | --- | --- | --- |
|  |  |  |  |  |  |  |
| GrimAge V1 | AUD Status | 1.52 | 0.55 | 2.76 | 6.14E-03 | * |
| GrimAge V2 | AUD Status | 1.90 | 0.59 | 3.20 | 1.55E-03 | * |
| CausAge | AUD Status | -0.23 | 0.56 | -0.42 | 0.68 |  |
| DamAge | AUD Status | -0.24 | 0.77 | -0.31 | 0.76 |  |
| AdaptAge | AUD Status | 1.38 | 0.94 | 1.47 | 0.14 |  |
|  |  |  |  |  |  |  |
| GrimAge V1 | Total Number of Drinks | 0.62 | 0.26 | 2.35 | 0.02 | . |
| GrimAge V2 | Total Number of Drinks | 0.72 | 0.28 | 2.52 | 0.01 | . |
| CausAge | Total Number of Drinks | 0.29 | 0.27 | 1.08 | 0.28 |  |
| DamAge | Total Number of Drinks | 0.15 | 0.37 | 0.40 | 0.69 |  |
| AdaptAge | Total Number of Drinks | -0.07 | 0.46 | -0.15 | 0.88 |  |
|  |  |  |  |  |  |  |
| GrimAge V1 | Number of Drinking Days | 0.71 | 0.39 | 1.80 | 0.07 |  |
| GrimAge V2 | Number of Drinking Days | 0.86 | 0.42 | 2.02 | 0.04 | . |
| CausAge | Number of Drinking Days | 0.16 | 0.41 | 0.38 | 0.70 |  |
| DamAge | Number of Drinking Days | -0.43 | 0.55 | -0.78 | 0.44 |  |
| AdaptAge | Number of Drinking Days | 0.23 | 0.67 | 0.34 | 0.74 |  |
|  |  |  |  |  |  |  |
| GrimAge V1 | Average Number of Drinks Per Day | 0.50 | 0.27 | 1.88 | 0.06 |  |
| GrimAge V2 | Average Number of Drinks Per Day | 0.55 | 0.29 | 1.89 | 0.06 |  |
| CausAge | Average Number of Drinks Per Day | 0.33 | 0.28 | 1.21 | 0.23 |  |
| DamAge | Average Number of Drinks Per Day | 0.12 | 0.38 | 0.32 | 0.75 |  |
| AdaptAge | Average Number of Drinks Per Day | 0.04 | 0.44 | 0.09 | 0.93 |  |
|  |  |  |  |  |  |  |
| GrimAge V1 | Number of Heavy Drinking Days | 1.11 | 0.33 | 3.39 | 8.18E-04 | ** |
| GrimAge V2 | Number of Heavy Drinking Days | 1.14 | 0.36 | 3.20 | 1.53E-03 | * |
| CausAge | Number of Heavy Drinking Days | 0.29 | 0.35 | 0.85 | 0.40 |  |
| DamAge | Number of Heavy Drinking Days | 0.32 | 0.47 | 0.69 | 0.49 |  |
| AdaptAge | Number of Heavy Drinking Days | -0.03 | 0.57 | -0.05 | 0.96 |  |
|  |  |  |  |  |  |  |
| GrimAge V1 | GGT | 0.51 | 0.22 | 2.25 | 0.03 | . |
| GrimAge V2 | GGT | 0.74 | 0.24 | 3.07 | 2.35E-03 | * |
| CausAge | GGT | -0.02 | 0.23 | -0.10 | 0.92 |  |
| DamAge | GGT | 0.15 | 0.32 | 0.47 | 0.64 |  |
| AdaptAge | GGT | -0.31 | 0.39 | -0.79 | 0.43 |  |
|  |  |  |  |  |  |  |
| GrimAge V1 | AST | 0.40 | 0.22 | 1.84 | 0.07 |  |
| GrimAge V2 | AST | 0.52 | 0.23 | 2.22 | 0.03 | . |
| CausAge | AST | 0.04 | 0.22 | 0.17 | 0.86 |  |
| DamAge | AST | 0.37 | 0.30 | 1.23 | 0.22 |  |
| AdaptAge | AST | -0.45 | 0.37 | -1.22 | 0.23 |  |
|  |  |  |  |  |  |  |
| GrimAge V1 | ALT | 0.27 | 0.22 | 1.24 | 0.22 |  |
| GrimAge V2 | ALT | 0.35 | 0.24 | 1.48 | 0.14 |  |
| CausAge | ALT | -0.04 | 0.23 | -0.18 | 0.86 |  |
| DamAge | ALT | 0.02 | 0.31 | 0.08 | 0.94 |  |
| AdaptAge | ALT | -0.29 | 0.38 | -0.76 | 0.45 |  |
|  |  |  |  |  |  |  |
| GrimAge V1 | CRP | 0.55 | 0.29 | 1.89 | 0.06 |  |
| GrimAge V2 | CRP | 0.85 | 0.32 | 2.69 | 7.76E-03 | * |
| CausAge | CRP | -0.60 | 0.29 | -2.02 | 0.04 | . |
| DamAge | CRP | 0.12 | 0.40 | 0.30 | 0.76 |  |
| AdaptAge | CRP | -0.01 | 0.48 | -0.03 | 0.98 |  |
|  |  |  |  |  |  |  |
| GrimAge V1 | A1C | 0.48 | 0.31 | 1.54 | 0.13 |  |
| GrimAge V2 | A1C | 0.63 | 0.33 | 1.90 | 0.06 |  |
| CausAge | A1C | -0.40 | 0.32 | -1.26 | 0.21 |  |
| DamAge | A1C | -0.51 | 0.44 | -1.16 | 0.25 |  |
| AdaptAge | A1C | 0.42 | 0.55 | 0.76 | 0.45 |  |
| Note: AUD = alcohol use disorder; GGT = gamma-glutamyl transferase; AST = aspartate aminotransferase; ALT = alanine aminotransferase; CRP = C-reactive protein; A1C = hemoglobin A1C; V1 = Version 1; V2 = Version 2. Heavy drinking days are defined as greater than or equal to 4 drinks a day for females and greater than or equal to 5 drinks a day for males. Significance codes are as follows: ‘***’ when P-value (P) < 0.0001, ‘**’ when P < 0.001, ‘*’ when P < 0.01, ‘.’ when P < 0.05. P-values were obtained through two sample t-tests. | | | | | | |

**Supplementary Table 4. Propensity score analysis-based associations of epigenetic clocks with alcohol consumption-based biomarkers in the optimally matched sample (N = 486).**

| Model | Alcohol Variable | β | SE | T | P-value | Significance |
| --- | --- | --- | --- | --- | --- | --- |
|  |  |  |  |  |  |  |
| GrimAge V1 | AUD Status | 1.96 | 0.46 | 4.23 | 2.76E-05 | *** |
| GrimAge V2 | AUD Status | 2.38 | 0.51 | 4.66 | 4.03E-06 | *** |
| CausAge | AUD Status | 0.29 | 0.40 | 0.72 | 0.47 |  |
| DamAge | AUD Status | 0.58 | 0.57 | 1.02 | 0.31 |  |
| AdaptAge | AUD Status | 0.61 | 0.73 | 0.83 | 0.41 |  |
|  |  |  |  |  |  |  |
| GrimAge V1 | Total Number of Drinks | 1.54 | 0.23 | 6.62 | 9.76E-11 | *** |
| GrimAge V2 | Total Number of Drinks | 1.68 | 0.26 | 6.57 | 1.30E-10 | *** |
| CausAge | Total Number of Drinks | 0.50 | 0.21 | 2.40 | 0.02 | . |
| DamAge | Total Number of Drinks | 0.26 | 0.30 | 0.89 | 0.38 |  |
| AdaptAge | Total Number of Drinks | 0.25 | 0.38 | 0.65 | 0.52 |  |
|  |  |  |  |  |  |  |
| GrimAge V1 | Number of Drinking Days | 0.78 | 0.29 | 2.67 | 7.77E-03 | * |
| GrimAge V2 | Number of Drinking Days | 0.74 | 0.32 | 2.32 | 0.02 | . |
| CausAge | Number of Drinking Days | 0.57 | 0.25 | 2.25 | 0.02 | . |
| DamAge | Number of Drinking Days | -0.21 | 0.36 | -0.58 | 0.56 |  |
| AdaptAge | Number of Drinking Days | 0.86 | 0.46 | 1.87 | 0.06 |  |
|  |  |  |  |  |  |  |
| GrimAge V1 | Average Number of Drinks Per Day | 1.44 | 0.24 | 5.93 | 6.21E-09 | *** |
| GrimAge V2 | Average Number of Drinks Per Day | 1.53 | 0.27 | 5.68 | 2.45E-08 | *** |
| CausAge | Average Number of Drinks Per Day | 0.51 | 0.22 | 2.37 | 0.02 | . |
| DamAge | Average Number of Drinks Per Day | 0.17 | 0.31 | 0.56 | 0.58 |  |
| AdaptAge | Average Number of Drinks Per Day | 0.36 | 0.39 | 0.91 | 0.36 |  |
|  |  |  |  |  |  |  |
| GrimAge V1 | Number of Heavy Drinking Days | 1.68 | 0.30 | 5.50 | 6.10E-08 | *** |
| GrimAge V2 | Number of Heavy Drinking Days | 1.87 | 0.33 | 5.58 | 4.07E-08 | *** |
| CausAge | Number of Heavy Drinking Days | 0.18 | 0.27 | 0.67 | 0.51 |  |
| DamAge | Number of Heavy Drinking Days | 0.35 | 0.38 | 0.90 | 0.37 |  |
| AdaptAge | Number of Heavy Drinking Days | 0.12 | 0.50 | 0.24 | 0.81 |  |
|  |  |  |  |  |  |  |
| GrimAge V1 | GGT | 0.45 | 0.19 | 2.39 | 0.02 | . |
| GrimAge V2 | GGT | 0.60 | 0.21 | 2.90 | 3.88E-03 | * |
| CausAge | GGT | 0.45 | 0.16 | 2.79 | 5.41E-03 | * |
| DamAge | GGT | 0.62 | 0.23 | 2.69 | 7.44E-03 | * |
| AdaptAge | GGT | 0.09 | 0.30 | 0.29 | 0.77 |  |
|  |  |  |  |  |  |  |
| GrimAge V1 | AST | 0.33 | 0.19 | 1.72 | 0.09 |  |
| GrimAge V2 | AST | 0.45 | 0.21 | 2.17 | 0.03 | . |
| CausAge | AST | 0.35 | 0.16 | 2.16 | 0.03 | . |
| DamAge | AST | 0.56 | 0.23 | 2.42 | 0.02 | . |
| AdaptAge | AST | -0.27 | 0.30 | -0.90 | 0.37 |  |
|  |  |  |  |  |  |  |
| GrimAge V1 | ALT | 0.22 | 0.19 | 1.17 | 0.24 |  |
| GrimAge V2 | ALT | 0.24 | 0.21 | 1.15 | 0.25 |  |
| CausAge | ALT | 0.37 | 0.16 | 2.28 | 0.02 | . |
| DamAge | ALT | 0.22 | 0.23 | 0.95 | 0.34 |  |
| AdaptAge | ALT | 0.20 | 0.30 | 0.68 | 0.50 |  |
|  |  |  |  |  |  |  |
| GrimAge V1 | CRP | 0.50 | 0.23 | 2.15 | 0.03 | . |
| GrimAge V2 | CRP | 0.70 | 0.26 | 2.73 | 6.71E-03 | * |
| CausAge | CRP | -0.10 | 0.20 | -0.52 | 0.61 |  |
| DamAge | CRP | 0.10 | 0.28 | 0.35 | 0.72 |  |
| AdaptAge | CRP | 0.23 | 0.36 | 0.65 | 0.52 |  |
|  |  |  |  |  |  |  |
| GrimAge V1 | A1C | -0.05 | 0.25 | -0.20 | 0.84 |  |
| GrimAge V2 | A1C | -0.09 | 0.27 | -0.32 | 0.75 |  |
| CausAge | A1C | -0.06 | 0.22 | -0.28 | 0.78 |  |
| DamAge | A1C | -0.38 | 0.31 | -1.21 | 0.23 |  |
| AdaptAge | A1C | 1.00 | 0.42 | 2.40 | 0.02 | . |
| Note: AUD = alcohol use disorder; GGT = gamma-glutamyl transferase; AST = aspartate aminotransferase; ALT = alanine aminotransferase; CRP = C-reactive protein; A1C = hemoglobin A1C; V1 = Version 1; V2 = Version 2. Heavy drinking days are defined as greater than or equal to 4 drinks a day for females and greater than or equal to 5 drinks a day for males. Significance codes are as follows: ‘***’ when P-value (P) < 0.0001, ‘**’ when P < 0.001, ‘*’ when P < 0.01, ‘.’ when P < 0.05. P-values were obtained through two sample t-tests. | | | | | | |
